# Unraveling the independent role of METTL3 in m6A modification and tumor progression in esophageal squamous cell carcinoma

**DOI:** 10.1038/s41598-024-64517-3

**Published:** 2024-07-04

**Authors:** Bin Du, Pu Wang, Lingyu Wei, Kai Qin, Zhen Pei, Jinping Zheng, Jia Wang

**Affiliations:** 1 Center of Healthy Aging, Changzhi Medical College, Changzhi, 047500 China; 2 Central Laboratory of Clinical Research, Heping Hospital Affiliated to Changzhi Medical College, Changzhi, 047500 China; 3 Department of Physiology, Changzhi Medical College, Changzhi, 047500 China

**Keywords:** METTL3, METTL14, Independent, Proliferation, Splicing, Bioinformatics, Oesophageal cancer

## Abstract

METTL3 and METTL14 are traditionally posited to assemble the m6A methyltransferase complex in a stoichiometric 1:1 ratio, modulating mRNA fate via m6A modifications. Nevertheless, recent investigations reveal inconsistent expression levels and prognostic significance of METTL3 and METTL14 across various tumor types, challenging their consistent functional engagement in neoplastic contexts. A pan-cancer analysis leveraging The Cancer Genome Atlas (TCGA) data has identified pronounced disparities in the expression patterns, functional roles, and correlations with tumor burden between METTL3 and METTL14, particularly in esophageal squamous cell carcinoma (ESCC). Knockdown experiments of METTL3 in EC109 cells markedly suppress cell proliferation both in vitro and in vivo, whereas METTL14 knockdown shows a comparatively muted effect on proliferation and does not significantly alter METTL3 protein levels. mRNA sequencing indicates that METTL3 singularly governs the expression of 1615 genes, with only 776 genes co-regulated with METTL14. Additionally, immunofluorescence co-localization studies suggest discrepancies in cellular localization between METTL3 and METTL14. High-performance liquid chromatography–mass spectrometry (HPLC–MS) analyses demonstrate that METTL3 uniquely associates with the Nop56p-linked pre-rRNA complex and mRNA splicing machinery, independent of METTL14. Preliminary bioinformatics and multi-omics investigations reveal that METTL3’s autonomous role in modulating tumor cell proliferation and its involvement in mRNA splicing are potentially pivotal molecular mechanisms. Our study lays both experimental and theoretical groundwork for a deeper understanding of the m6A methyltransferase complex and the development of targeted tumor therapies focusing on METTL3.

## Introduction

METTL3 serves as the catalytic subunit of the N6-adenosine methyltransferase complex and, in conjunction with METTL14, comprises the “writer” complex essential for catalyzing m6A modifications on mRNA^1^. The presence of m6A modifications on mRNA facilitates their recognition by “reader” proteins, thereby enhancing the processes of mRNA translation or degradation^2^. m6A modifications can also be reversed by “eraser” enzymes. The METTL3–METTL14-mediated m6A modification plays a pivotal role in regulating cellular proliferation, differentiation, and responses to various stresses. In recent years, mRNA m6A methylation mediated by the METTL3–METTL14 complex has offered a novel paradigm for elucidating the mechanisms underlying cancer progression. Numerous studies have demonstrated that the upregulation of METTL3, frequently observed in tumors, promotes tumor cell survival, proliferation, self-renewal, metastasis, and drug resistance by modulating mRNA metabolism^3^.

Elevated expression of METTL3/METTL14 has been significantly correlated with the advancement and adverse prognosis of various cancers^4^. In acute myeloid leukemia, the overexpression of METTL3/METTL14 enhances the stability and translation efficiency of c-myc via m6A modifications, thereby accelerating cancer progression^5^. In breast cancer, the METTL3-METTL14 complex upregulates the expression of genes like Bcl-2 and CXCR4 through m6A-dependent mechanisms, thereby fostering breast cancer growth and metastasis^6^. Nevertheless, in certain cancers, METTL3 and METTL14 demonstrate divergent effects on tumor progression. Knockdown of METTL3 has been observed to alter the malignant phenotype across various tumor types, whereas multiple studies have indicated that elevated expression of METTL14 inhibits tumor proliferation and migration via m6A-dependent mechanisms. These contrasting prognostic impacts have been documented in colorectal, bladder, lung, gastric, and other cancers^7,8^.

Additional research has demonstrated that the regulatory effect of METTL3 on downstream genes may operate independently of METTL14. For instance, in ovarian cancer, METTL3 shows a significant association with prognosis; however, the knockdown of METTL14 does not inhibit the clonogenic formation capacity of TOV-112D cells. Moreover, the expression of oncogenes such as EIF3C, AXL, CSF1, and FZD10 is solely modulated by METTL3, underscoring its unique regulatory role^9^. Similarly, in esophageal squamous cell carcinoma (ESCC), METTL3 and METTL14 demonstrate distinct functional roles. Specifically, the modulation of miR-99a-5p by METTL14 occurs independently of METTL3, highlighting differential regulatory pathways within this cancer type^10,11^.

The findings from the referenced studies elucidate that while METTL3 and METTL14, integral components of the m6A writer complex, collaboratively influence m6A modifications in cells, they display marked disparities in their influence on downstream gene regulation in tumor cells, either directly or indirectly. The distinct mechanisms by which METTL14 regulates genes independently of METTL3 pose critical questions for future m6A research. In our current study, employing a pan-cancer analytical approach, we developed METTL3 and METTL14 knockdown EC109 esophageal cancer cell lines to explore the impact of gene silencing on cellular proliferation. Additionally, comprehensive transcriptomic and proteomic analyses were conducted to delineate the intricate molecular mechanisms involved.

## Materials and methods

### Pan-cancer analysis

We retrieved RNA-sequencing expression profiles at level 3 and associated clinical data for different cancer types from the Cancer Genome Atlas (TCGA) database, accessible via the website https://portal.gdc.com. Subsequent statistical analysis was carried out using R version 4.0.3 software, provided by the R Foundation for Statistical Computing, based in Vienna, Austria, with a significance threshold set at P-value < 0.05.

All the analyses and implementations of the aforementioned methods were conducted using R version 4.0.3, which is supported by the R Foundation for Statistical Computing (2020). We utilized the ggplot2 package for creating clear and concise graphics, and the pheatmap package for generating informative heatmaps to visualize our data.

### Correlation map

We applied Spearman’s correlation analysis to quantify the relationship between non-normally distributed quantitative variables. For determining statistical significance, we adopted a P-value cutoff of less than 0.05, with results achieving P < 0.05 being considered statistically noteworthy.

### Pathways clustering

The clustering analysis were performed at Metascape (https://metascape.org/)^12^.

### Transfection

The EC109 cells utilized in the experiments were obtained from the Cell Resource Center, Peking Union Medical College (PCRC). The cells were cultured in high-glucose DMEM medium, incubated at 37 °C in a 5% CO2 incubator, with the medium containing 10% serum. Lentiviral vector targeting METTL3 and METTL14 were purchased from Bocui technology (Shanxi China). The plasmid pLKO.1-U6-EF1a-mcherry(copGFP)-T2A-Neo(puro) were was constructed to knockdown the expression of the target gene. After virus infection, G418 or puromycin is used for selection. The sh-RNA sequence used for knockdown is as follows:

- sh-METTL14-1: AAGGATGAGTTAATAGCTAAACTCGAGTTTAGCTATTAACTCATCCTT
- sh-METTL14-2:
- TGGTGCCGTGTTAAATAGCAACTCGAGTTGCTATTTAACACGGCACCA
- sh-METTL3-1: CTGCAAGTATGTTCACTATGACTCGAGTCATAGTGAACATACTTGCAG
- sh-METTL3-2:
- AGGAGCCAGCCAAGAAATCAACTCGAGTTGATTTCTTGGCTGGCTCCT

### Western blot

To isolate total protein from esophageal carcinoma cells, RIPA lysis buffer (Beyotime, Shanghai, China) was utilized. Following the extraction process, the membranes were washed six times with TBST for five minutes each. Subsequently, they were incubated with HRP-conjugated secondary antibodies specific to rabbit or mouse IgG at a dilution of 1:5000, at 4 °C for a 12-h period. Protein levels were detected using the ChemiDOC™ XRS + imaging system (Bio-Rad). For quantification of protein bands, Olympus Image-Pro Plus software was employed. The immunoblots were captured digitally using said imaging equipment. The antibodies applied in the western blot analysis included: anti-METTL3 (Abcam, ab195352), anti-METTL14 (Abcam, ab300104), anti-beta-actin (Abcam, ab8226), anti-HNRNPM (Proteintech, 26897-1-AP), anti-U2AF1 (Propteintech, 10334-1-AP), Goat Anti-Rabbit IgG H&L (HRP) (Abcam, ab205718), and Goat Anti-Mouse IgG H&L (HRP) (Abcam, ab205719). Imaging was exclusively performed using a chemiluminescence module. In Fig. 2E, for which more comprehensive original data cannot be provided. This is due to the section above 50 kDa being excised for use in other experiments during the experimental process.

### Clonal formation assay

EC109 cells underwent an initial trypsinization process, followed by cell counting. Then, 1000 cells were seeded into each well of a 6-well plate. These cells were cultured in a humidified incubator set at 37 °C with 5% CO2 for a duration of 10 days, which allowed for colony formation. After the colonies had formed, we discarded the culture medium and gently washed the cells with PBS. For fixation, formaldehyde was applied, after which the cells were stained using crystal violet solution (Sangon Biotech, E607309) to visualize the colonies.

### Tumor-bearing mouse models

The conduct of mouse-related experiments was approved by the animal ethics committee at Changzhi Medical College, ensuring adherence to all pertinent guidelines and regulations. We ensured compliance with the ARRIVE guidelines throughout the execution of mouse experiments. All mice were housed in specific pathogen-free conditions, with regulations on temperature and humidity maintained, alongside a consistent 12-h light/dark cycle. Mice consumed soya-free laboratory chow and had access to tap water ad libitum. EC109 cells were subcutaneously injected into the left flank of 5-week-old female BALB/c nude mice. Subsequent to the injection, tumor size and survival rate were monitored bi-daily. Once the tumors attained a volume of 300 mm^3^, the animals were humanely euthanized through CO2 inhalation in concordance with ethical practices.

### Apoptosis analysis

Cells undergoing routine culture were digested with EDTA-free trypsin to dissociate them into a single cell suspension after reaching confluence. The detached cells were then suspended in PBS, and subsequently stained with Annexin V-FITC and propidium iodide (PI) (Beyotime, C1062S) for apoptosis analysis. This staining procedure took place in a dark environment for 20 min to prevent photobleaching of the fluorescent dyes. Immediately following the incubation period, the stained cells were analyzed using a flow cytometer for apoptosis detection.

### mRNA-seq

Total RNA was extracted employing the Trizol reagent (Thermo Fisher) in strict accordance with the instructions provided by the manufacturer. The isolated RNA was then utilized to construct an RNA-seq library using the NEBNext® Ultra™ II Directional RNA Library Prep Kit for Illumina (NEB). Sequencing was performed for both non-immunoprecipitated (initial sample) and m6A-immunoprecipitated RNA (m6A IP sample), involving 150 bp paired-end sequencing on the Illumina HiSeq platform. The sequencing data obtained was of high quality, with a Q30 score assessment conducted to ensure reliability. Trimming of 3ʹ adaptors and removal of low-quality reads were executed using cutadapt software (version 1.9.3). Cleaned reads were aligned to the reference human genome (UCSC HG19) with the aid of the Hisat2 software (version 2.0.4).

### Immunofluorescence staining

SCC cells were cultured on coverslips, washed with PBS, and fixed with 4% paraformaldehyde. Post-fixation, they were blocked with bovine serum albumin for 60 min and incubated with anti-METTL14 and anti-METTL3 antibodies overnight at 4 °C. Following three PBS washes, cells were incubated for 60 min with Alexa Fluor® 488-conjugated goat anti-rabbit IgG (Abcam, ab150077, 1:1000) and Alexa Fluor® 594-conjugated goat anti-mouse IgG (Abcam, ab150116, 1:1000). After mounting with Sigma’s mounting medium (F6057), cells were observed under an Olympus IX73 microscope. ESCC tissue microarrays were purchased from Shanghai Outdo Biotech in Shanghai, China (HEsoS060CS01). Experiments using human samples have been approved by the Human Ethics Committee.

The immunofluorescence colocalization analysis was conducted using Olympus analysis software (cellSens Dimension). Initially, pseudocolor was added to the fluorescence images, followed by multichannel fusion. Deconvolution was then performed using the nearest neighbor algorithm. Finally, the colocalization analysis module was utilized for analysis.

### LC–MS/MS analysis

The phosphoproteomic study was conducted using a Thermo Fisher Scientific Easy nLC 1200 system, paired with a Q Exactive HF-X mass spectrometer. Phopeptides were loaded onto a custom-packed column with buffer A and eluted over a 110-min period at a flow rate of 300 nL/min, utilizing a linear increase of buffer B covering 2–40%. Mass spectra were collected in a range from m/z 350 to m/z 1800, at a resolution of 60,000 at m/z 200, and a maximum injection time of 50 ms per scan. The top 15 most intense precursor ions from the MS scan were selected for higher-energy collisional dissociation (HCD) MS/MS with an isolation window of 1.6 Th, dynamic exclusion for 30 s, and a resolution of 15,000 at m/z 200.

### Statistics

In this study, comparisons between two groups were conducted using Student’s t-test, and comparisons among multiple groups were performed via one-way ANOVA. The results are presented as mean ± standard deviation.

### Statement

All methods were carried out in accordance with relevant guidelines and regulations. All experimental protocols were approved by Changzhi medical college licensing committee. We confirm that informed consent was obtained from all subjects and/or their legal guardians.

## Results

### Differential expression and functional disparities of METTL3 and METTL14 in ESCC

**Figure 1:**
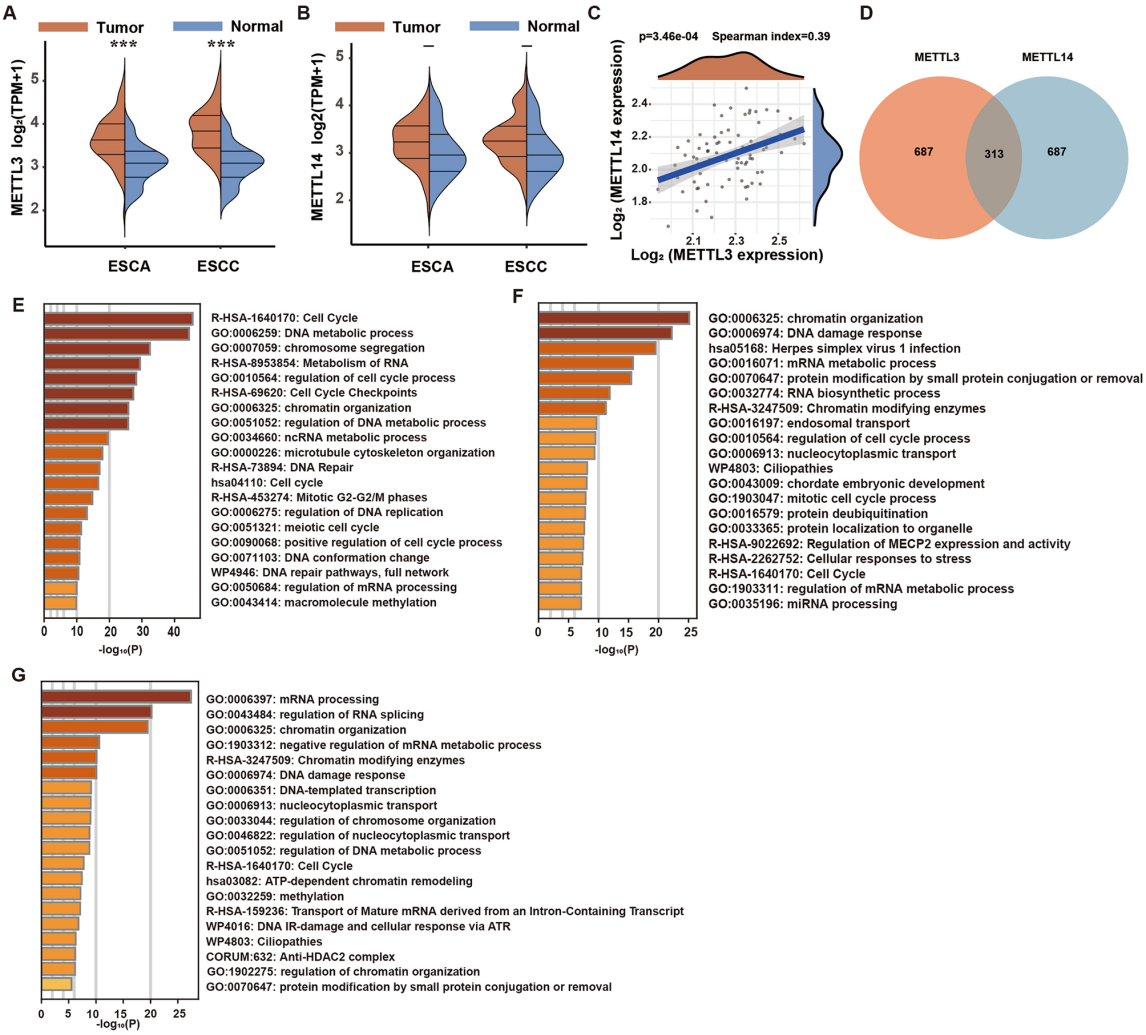
Bioinformatics analysis of the differential expression of METTL3 and METTL14 in ESCC. (**A**) Expression of METTL3 in ESCA, ESCC, and normal tissues. (**B**) Expression of METTL14 in ESCA, ESCC, and normal tissues. (**C**) Correlation analysis of METTL3 and METTL14 expression in ESCC. (**D**) Venn analysis of METTL3 and METTL14 co-expressed genes. (**E**) Clustering analysis of genes that are only positively correlated with METTL3 expression. (**F**) Clustering analysis of genes positively correlated with both METTL3 and METTL14 expression. (**G**) Clustering analysis of genes only positively correlated with METTL13 expression; *P < 0.05, **P < 0.01, ***P < 0.001.

Initially, we interrogated the TCGA database for prevalent tumors, observing distinct mRNA expression patterns for METTL3 and METTL14, which typically form a 1:1 complex in healthy tissues. Notably, METTL3 was significantly upregulated across various tumors, especially within the digestive system (e.g., ESCA, STAD, LIHC, COAD, READ), as depicted in Fig. S1A. In contrast, METTL14’s expression was generally reduced compared to normal tissues, as shown in Fig. S1B. Interestingly, in some cancers, predictive models incorporating only METTL3 and METTL14, constructed using Cox or Lasso algorithms, effectively generated prognostic signatures, where their contributions to the risk scores were diametrically opposed (ESCC: Risk score = (0.1783) × METTL3 + (− 0.4982) × METTL14), as illustrated in Fig. S2. Further analysis revealed significant disparities in tumor mutation burden and microsatellite stability between METTL3 and METTL14, shown in Fig. S3A,B, respectively. Subsequent investigations into ESCA and ESCC confirmed significant alterations in mRNA expression for these genes. In ESCA, METTL3 levels were markedly higher than in normal tissue (Fig. 1A), whereas METTL14 levels did not differ significantly (Fig. 1B). A correlation study between METTL3 and METTL14 mRNA expressions highlighted a modest positive correlation (Spearman coefficient = 0.39), as detailed in Fig. 1C. Additionally, we analyzed the top 1000 genes that were positively correlated with each gene. A Venn diagram showed that only 313 of these genes were common between METTL3 and METTL14 (Fig. 1D). Cluster analysis of METTL3-specific positively correlated genes showed significant enrichment in cell cycle, DNA metabolic processes, and chromosome segregation pathways (Fig. 1E), whereas genes co-positively correlated with both displayed notable enrichment in chromatin organization, DNA damage, and mRNA metabolism pathways (Fig. 1F). Contrastingly, the enrichment pathways for METTL14-specific genes included chromatin organization, DNA repair, and mRNA splicing (Fig. 1G). Despite the normal 1:1 ratio formation between METTL3 and METTL14 in healthy cells, gene mutations, abnormal regulation, or changes in environmental factors during the tumor development process may disrupt this balance, resulting in differences in their expression and function.

### Knockdown of METTL3 or METTL14 has different effects on cell growth

**Figure 2:**
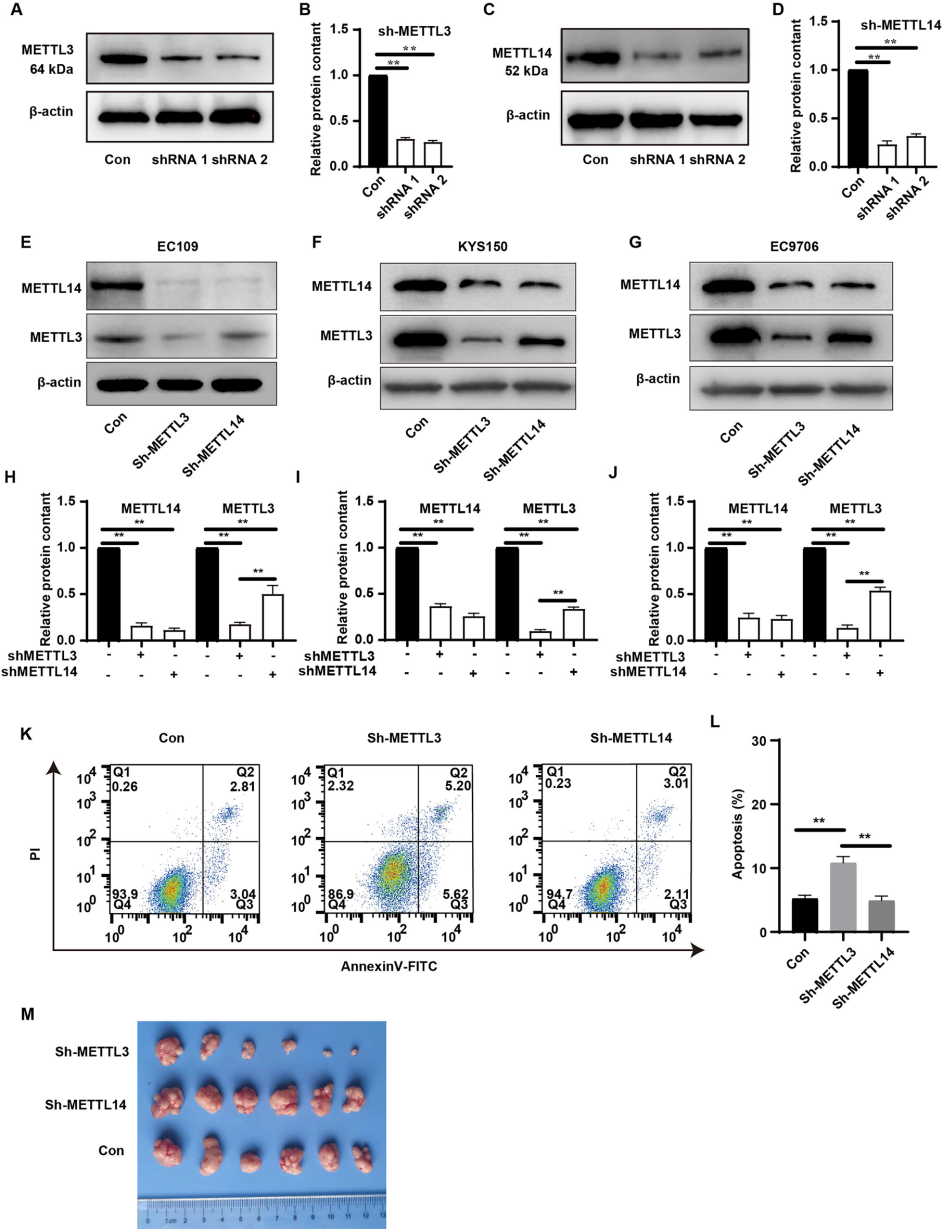
(**A**) Using two unique siRNAs to knock down METTL3 expression in EC109 cells and analyzing the grayscale values of Western blot bands (**B**). (**C**) Using two unique siRNAs to knock down METTL14 expression in EC109 cells and analyzing the grayscale values of Western blot bands (**D**). (**E–G**) Transfecting EC109, KYS150, EC9706 cells with two types of shRNAs targeting METTL3 or METTL14, measuring the protein levels of METTL3 and METTL14 and performing grayscale analysis (**H–J**), and assessing the natural apoptosis rate in EC109 cells (**K,L**); (**M**) Constructing stable cell lines with knocked down METTL3 or METTL14, and evaluating the tumorigenic capacity of different cell lines using a subcutaneous tumor model. *P < 0.05, **P < 0.01.

Initially, we effectively knocked down the expression of METTL3 (Fig. 2A,B) and METTL14 (Fig. 2C,D) in EC109 cells using two distinct siRNAs for each gene. Subsequently, to achieve a more efficient knockdown of METTL3 and METTL14, in the following experiments, we co-transfected the cells with two siRNAs targeting the same gene; this was done separately in EC109, KYS150, and EC9706 cells to reduce the expression of the target genes. Surprisingly, knockdown of METTL3 significantly reduced the protein levels of both METTL3 and METTL14, whereas knockdown of METTL14 did not effectively suppress the expression of METTL3 (Fig. 2E–J), with varying degrees of this phenomenon observed across all three cell lines. Further experiments revealed that in EC109 cells, knockdown of METTL3 significantly inhibited cell proliferation, whereas knockdown of METTL14 had no substantial impact on the growth and clonogenic ability of EC109 cells (Fig. S4). Flow cytometry analysis showed that cells with METTL3 knockdown exhibited a higher baseline apoptosis rate (Fig. 2K,L). Additionally, a subcutaneous tumor model in nude mice demonstrated that loss of METTL3 significantly inhibited the tumorigenic ability of EC109 cells, while loss of METTL14 did not significantly affect tumor growth (Fig. 2M). In summary, our in vitro and in vivo experiments have elucidated the differing roles of METTL3 and METTL14 proteins in tumor cell proliferation capability.

### Transcriptome sequencing of METTL3 or METTL14 knockdown cells

**Figure 3:**
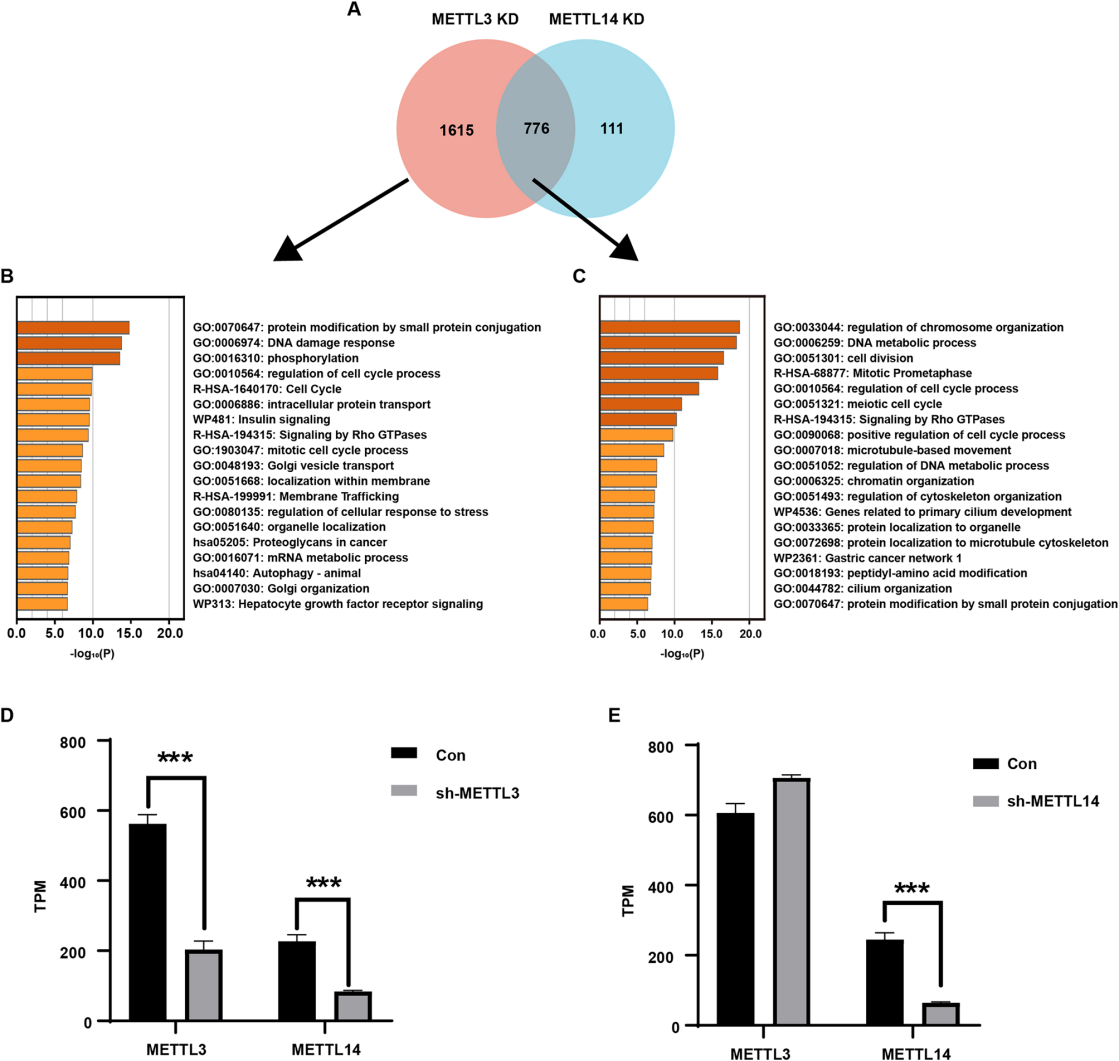
mRNA-seq analysis of changes in mRNA expression profiles in METTL3 or METTL14 knockdown cells. (**A**) Venn analysis of downregulated genes relative to wild-type cells after METTL3 and METTL14 knockdown. (**B**) Cluster analysis of genes independently regulated by METTL3 without the involvement of METTL14. (**C**) Cluster analysis of genes co-regulated by METTL3 and METTL14. (**D**) mRNA expression levels of METTL3 and METTL14 after METTL3 knockdown. (**E**) mRNA expression levels of METTL3 and METTL14 after METTL14 knockdown. ***P < 0.001.

Through mRNA-seq, we observed the impact of METTL3 or METTL14 knockdown on the mRNA expression profile of EC109 cells. After METTL3 knockdown, 2392 genes were downregulated and 396 genes were upregulated, while after METTL14 knockdown, 887 downregulated genes and 132 upregulated genes detected (Table S1). Venn analysis revealed that among the downregulated genes caused by METTL3 and METTL14 knockdown, 776 genes were co-regulated by both METTL3 and METTL14, 1615 genes were independently regulated by METTL3, and only 111 genes were independently regulated by METTL14 (Fig. 3A). Cluster analysis showed that METTL3 and METTL14 co-regulated pathways such as chromosome organization, DNA metabolism, cell division, and Rho GTPases (Fig. 3B). Pathways involved in independently regulated by METTL3 were protein modification by small protein conjugation, DNA damage response, phosphorylation, regulation of cell cycle process, Golgi vesicle transport, regulation of cellular response to stress, and autophagy etc. (Fig. 3C). The mRNA-seq data further confirmed that knockdown of METTL3 significantly reduced the expression of METTL14, while knockdown of METTL14 had no effect on the mRNA expression of METTL3 (Fig. 3D,E).

### Proteomic identification binding partners of METTL3 independent of METTL14

**Figure 4:**
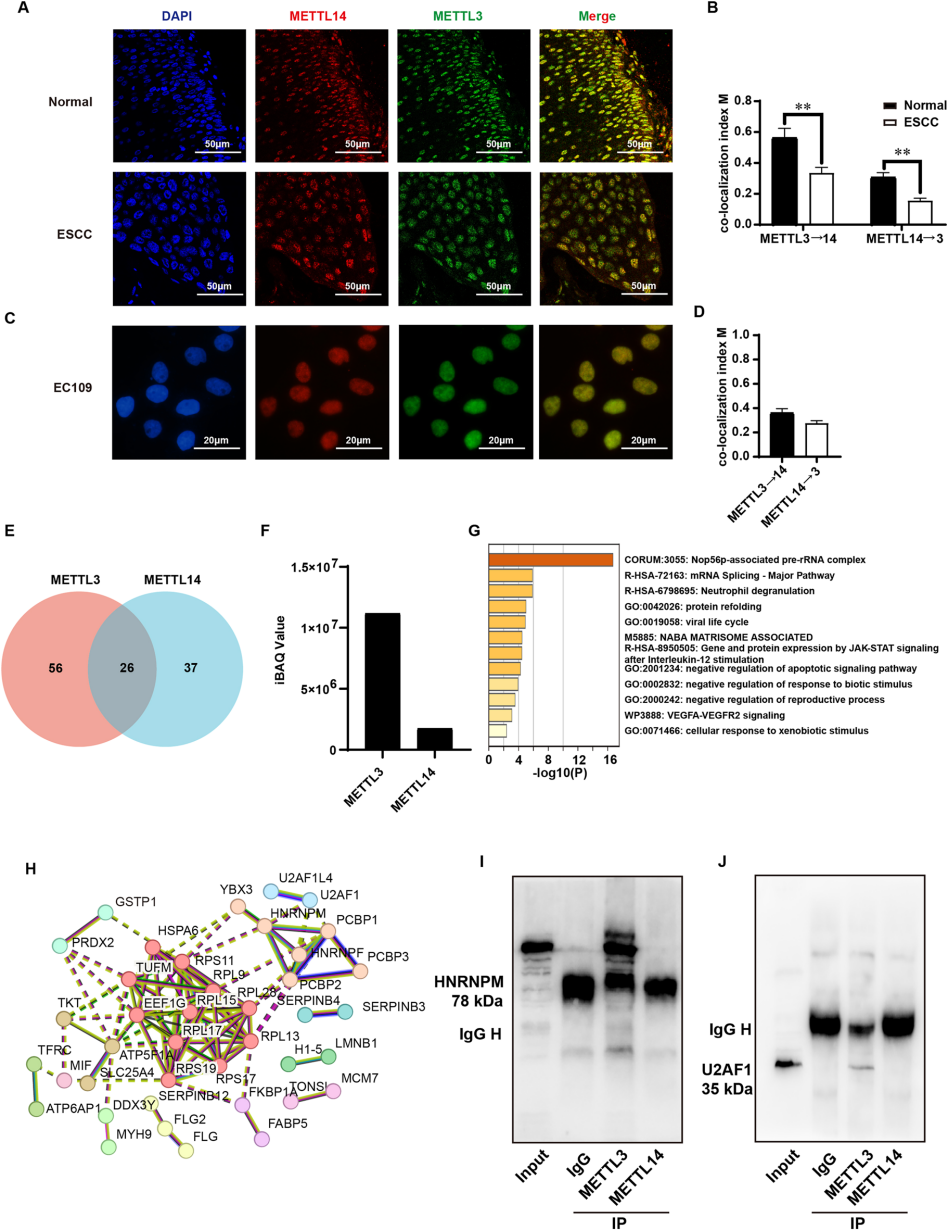
Proteomic identification of METTL3 and METTL14 binding partners. (**A**) Immunofluorescence staining of METTL3 and METTL14 in ESCC tissues using anti-METTL3 and anti-METTL14 antibodies; (**B**) the co-localization index M of METTL3 and METTL14 in tissues. (**C**) Immunofluorescence staining of METTL3 and METTL14 in EC109 cells using anti-METTL3 and anti-METTL14 antibodies; (**D**) the co-localization index M of METTL3 and METTL14 in EC109 cell; (**E**) Venn analysis of proteins bound by METTL3 and METTL14. (**F**) The abundance of METTL3 and METLT14 pulled down by anti-METTL3 antibody. (**G**) Cluster analysis of the binding proteins of METTL3. (**H**) Network analysis of METTL3 binding proteins. (**I**) Protein immunoprecipitation using antibodies against METTL3 and METTL14 to detect the protein HNRNPM and U2FA1 (**J**). **P < 0.01.

Based on the aforementioned study, we further hypothesize that METTL3 has independent functions from METTL14 in esophageal cancer cells (EC109), possibly through the interaction with other complexes. Therefore, we first examined the subcellular localization of METTL3 and METTL14 through co-immunofluorescence staining in tumor tissues (Fig. 4A,B) and EC109 cells (Fig. 4C,D). The results showed that neither METTL3 nor METTL14 exhibited cytoplasmic localization, and their nuclear localization exhibited substantial deviation, particularly in tumor tissues. It further reinforced our hypothesis that METTL3 has independent binding partners from METTL14. Subsequently, we performed co-immunoprecipitation using anti-METTL3 and anti-METTL14 antibodies followed by HPLC–MS identification of the pulled-down proteins. The identification results revealed that METTL3 has binding partners independent of METTL14, including 56 proteins (Fig. 4E). Furthermore, based on the iBAQ values, we observed that the abundance of METTL14 pulled down by the anti-METTL3 antibody was only about 20% of the abundance of METTL3 (Fig. 4F). Cluster analysis revealed that METTL3’s independent binding partners mainly involved the Nop56p-associated pre-rRNA complex, mRNA splicing complex, and other signaling pathways such as neutrophil degranulation and protein refolding (Fig. 4G). The protein network analysis was shown in Fig. 4H. We detected differences in the binding of the splicing factor HNRNPM and U2FA1 protein by METTL3/METTL14 through protein immunoprecipitation (Fig. 4I–J).

## Discussion

In normal cells, METTL3 and METTL14 interact to form a heterodimer with a stoichiometry of 1:1, which facilitates the methylation of RNA via m6A modifications. A pan-cancer analysis leveraging data from TCGA revealed that, in contrast to normal tissues, mRNA expression levels of METTL3 are significantly upregulated in tumor tissues, whereas the expression levels of METTL14 remain unchanged (Fig. S1). Furthermore, co-immunoprecipitation studies in tumor cells have demonstrated that the binding affinity of METTL3 for METTL14 is significantly lower than that of METTL14 for METTL3. This disparity suggests an excess of METTL3 protein abundance relative to METTL14, with the surplus METTL3 not participating in or forming the methyltransferase ‘writer’ complex with METTL14^13^. In normal cells, the loss of either METTL3 or METTL14 subunit leads to the degradation of the other subunit^14,15^. However, in tumor cells, the loss of METTL3 can decrease the protein expression of METTL14, while knockdown of METTL14 does not significantly affect METTL3 (Fig. 2E–G)^16^. This indicates that the protein stability of METTL3 and METTL14 is no longer mutually dependent.

METTL3 is significantly upregulated in various tumors and regulates signaling pathways such as p53, Ras/Raf/ERK, and Wnt/β-catenin in an m6A-dependent manner, promoting the proliferation and metastasis of liver cancer cells^8^. On the other hand, overexpression of METTL14 in cells can inhibit the EGFR/PI3K/AKT signaling pathway in an m6A methylation-dependent manner, thereby suppressing tumor metastasis^17^. METTL14 also regulates the glycolytic pathway and inhibits tumor proliferation through the USP48-SIRT6 pathway^7^. These findings are consistent with our bioinformatics analysis results (Fig. 1). When we performed survival analysis using METTL3 and METTL14 as a gene set, we found that METTL3 and METTL14 contributed completely opposite prognostic risk values in liver cancer (Fig. S2C). Although METTL3 and METTL14 have different prognostic values as components of the m6A complex, overall, they are still associated with poor prognosis in tumors.

To investigate the underlying molecular mechanisms, we also conducted preliminary studies using RNA-seq. We found that METTL3, independent of METTL14, regulates pathways such as Cell Cycle and DNA damage response (Fig. 3B). The regulatory mechanism of METTL3 independent of METTL14 has been rarely reported. Some studies suggest that cytoplasmic localized METTL3 can directly bind to the eukaryotic translation initiation factor eIF3h, independent of METTL14. The amino acid residue 155A of METTL3 is a key site for its direct interaction with eIF3h, forming dense polyribosomes. Ribosome-localized METTL3 enhances mRNA translation through a gap-dependent mechanism and plays an important role in the progression of lung cancer^18^. A similar mechanism has been observed in chronic myeloid leukemia^19^. However, in our study, both at the cellular level and in tumor tissues, METTL3 did not exhibit cytoplasmic localization (Fig. 4A,C), suggesting that the METTL3-independent function from METTL14 occurs within the nucleus, at least under our experimental conditions.

Within the nucleus, the association of METTL3–METTL14 with the transcriptional complex is an important mechanism for efficient mRNA modification by the “writer” complex. In normal cells, METTL3 and METTL14 form a complex that faithfully associates with the transcriptional complex. METTL14 binds to trimethylated histone H3K36me3^13^, allowing the coupling of METTL3-METTL14 with the transcriptional complex, along with specific transcription factors, to link transcription with m6A modification on RNA^20^. Their binding sites on DNA are highly consistent. However, during cellular senescence, there is only 13% overlap in the binding of METTL3 and METTL14 on DNA sequences^10,11^. This suggests that, under specific conditions, the binding of METTL3 and METTL14 to DNA is not faithful. In acute myeloid leukemia, METTL3 and METTL14 exhibit binding to transcription start sites, but the binding sites of METTL3 and METTL14 are almost entirely distinct^21,22^. Researchers speculate that the differential regulatory patterns of METTL3 and METTL14 may be due to differences in the pathways by which the “writer” complex binds to mRNA. However, in our study, with EC109 esophageal squamous cell carcinoma cells, we found significant differences in the binding partners of METTL3 and METTL14 (Fig. 4). Among the shared binding partners of METTL3 and METTL14, we identified histone and transcription-related proteins (such as TAF15), which confirms the association between the m6A modification process and transcription. However, we also found that METTL3 binds to many partners independent of METTL14. This suggests that the large differences in DNA binding sites observed in senescent or cancer cells may be due to partial dissociation of the METTL3-METTL14 complex or redundancy in the protein abundance of METTL3 compared to METTL14.

We also found that METTL3 independently binds to proteins associated with mRNA splicing, such as U2AF1, HNRNPM, PCBP1, suggesting that METTL3 may regulate cellular processes through binding to the mRNA splicing complex. m6A reader proteins highly associated with m6A modifications, such as HNRNPA2B1^23^, HNRNPC^24^, YTHDC1^25,26^, are known to play a widespread role in regulating m6A-dependent alternative splicing. In nascent RNA, 10% of m6A modification sites are located within introns or exons near the 5ʹ splice site. Rapid depletion of METTL3 eliminates existing m6A modification sites within introns and alters the original splicing pattern^27^. However, in our study, the splicing-associated proteins bound by METTL3, such as U2AF1^28^, HNRNPF^29^, PCBP2^30^, all have binding capabilities at the 3ʹ end. The role of METTL3 in splicing proteins may not depend on its m6A-writing ability, similar to its binding to eIF3h^18^. It is worth noting that proteins like HNRNPF and PCBP2 have binding capabilities for G-rich or C-rich sequences. Further research is needed to investigate the binding and function of METTL3 with splicing proteins.

In conclusion, through bioinformatic, transcriptomic, and proteomic approaches, we reported the differences in expression, prognostic value, regulatory mechanisms, and distribution of METTL3 and METTL14 in tumors. However, there are still many questions that require further investigation. For example, how much redundancy exists between METTL3 and METTL14? Does the METTL3 functionality independent of METTL14 depend on its m6A-writing ability? What is the mechanism by which METTL3, independent of METTL14, binds to different complexes? Does it rely on RNA or recruitment by other proteins? These aspects need to be further investigated.

### Supplementary Information

Supplementary Information.

Supplementary Figure S1.

Supplementary Figure S2.

Supplementary Figure S3.

Supplementary Figure S4.

Supplementary Table S1.

## Data Availability

The raw data of mRNA-seq has been uploaded to the GEO database (GSE254232), and the proteomic data has been uploaded to the PRIDE database (PXD048886).
