# Supplementary material for: Unraveling the independent role of METTL3 in m6A modification and tumor progression in esophageal squamous cell carcinoma
Source: Sci Rep. 2024 Jul 4;14:15398. doi: 10.1038/s41598-024-64517-3 (PMC11224396; doi:10.1038/s41598-024-64517-3)
Supplement: Supplementary file 1 — Supplementary Information. [file 41598_2024_64517_MOESM1_ESM.pptx]

## Slide 1
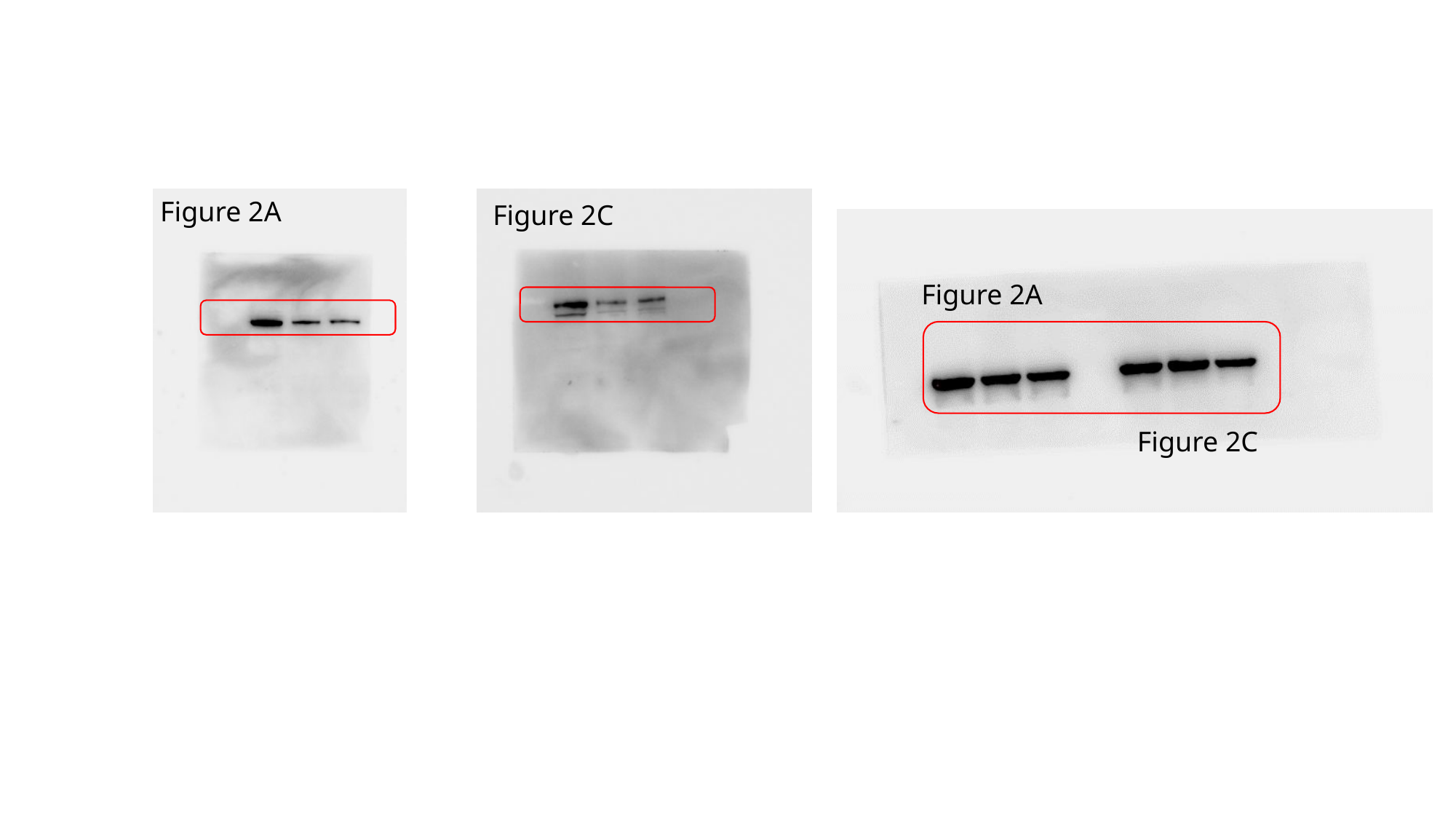

Figure 2A
Figure 2C
Figure 2A
Figure 2C

## Slide 2
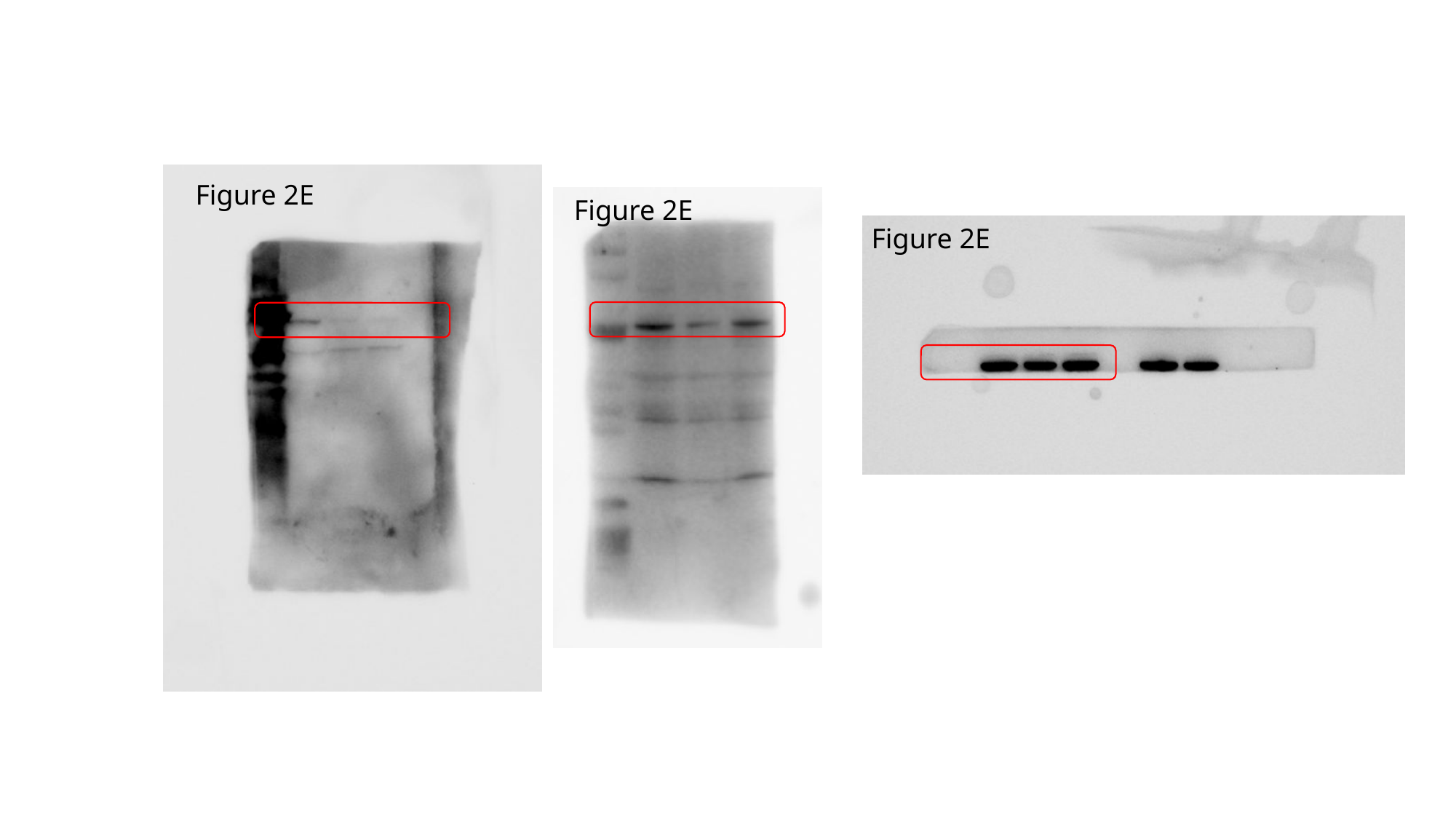

Figure 2E
Figure 2E
Figure 2E

## Slide 3
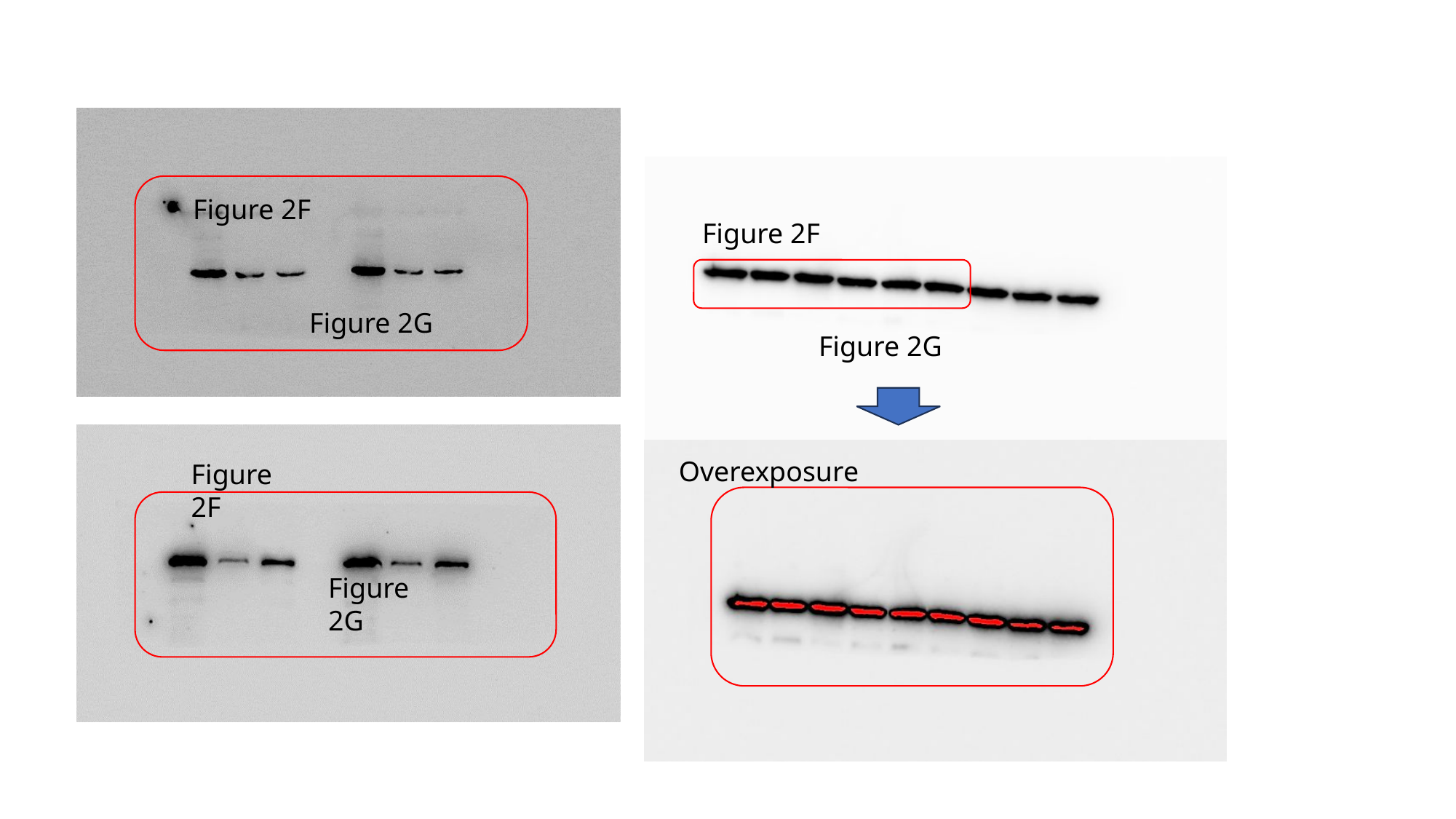

Figure 2F
Figure 2F
Figure 2G
Figure 2G
Overexposure
Figure 2F
Figure 2G

## Slide 4
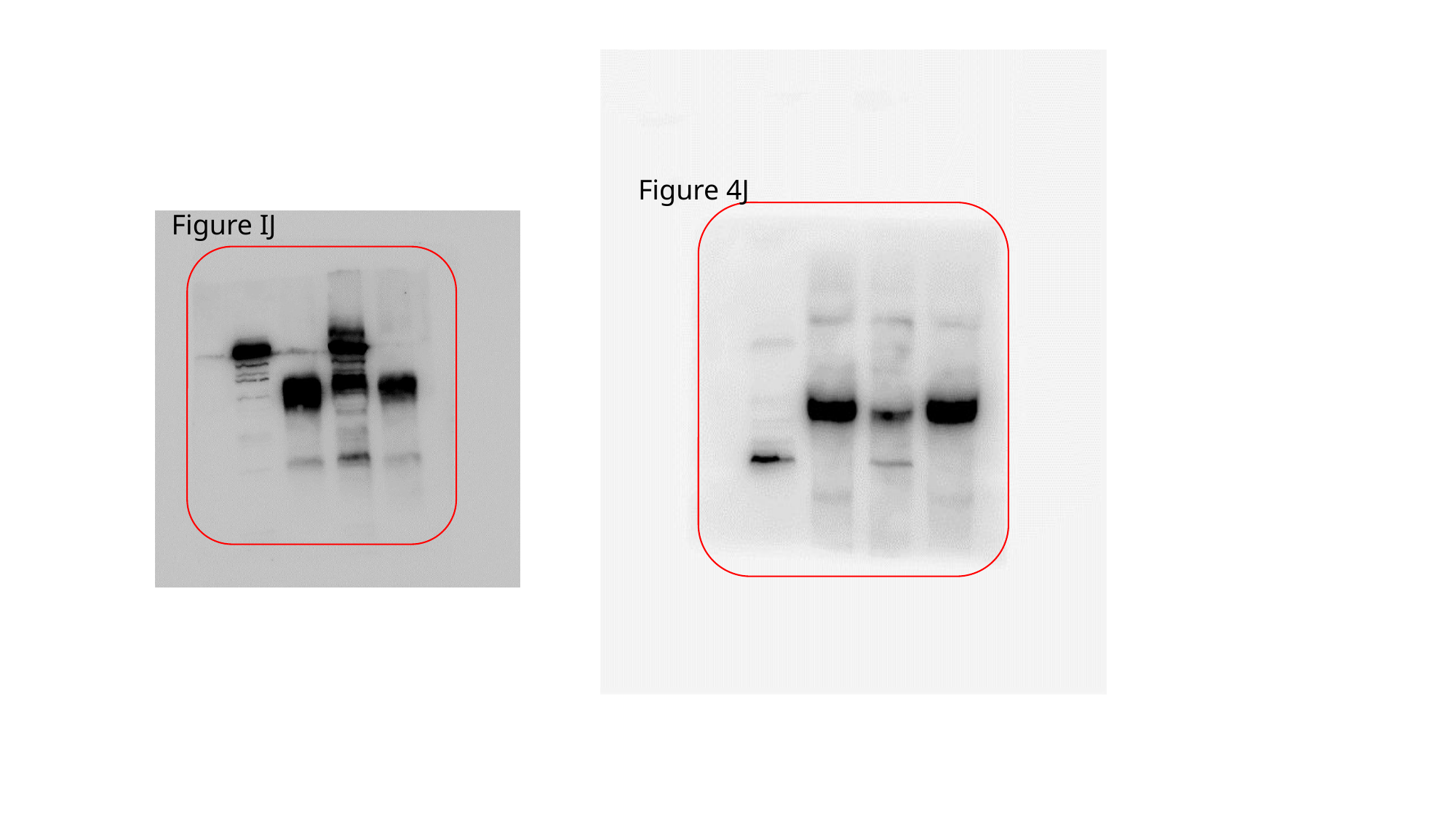

Figure 4J
Figure IJ
