# Supplementary figures and images for: Unraveling the independent role of METTL3 in m6A modification and tumor progression in esophageal squamous cell carcinoma
Source: Sci Rep. 2024 Jul 4;14:15398. doi: 10.1038/s41598-024-64517-3 (PMC11224396; doi:10.1038/s41598-024-64517-3)

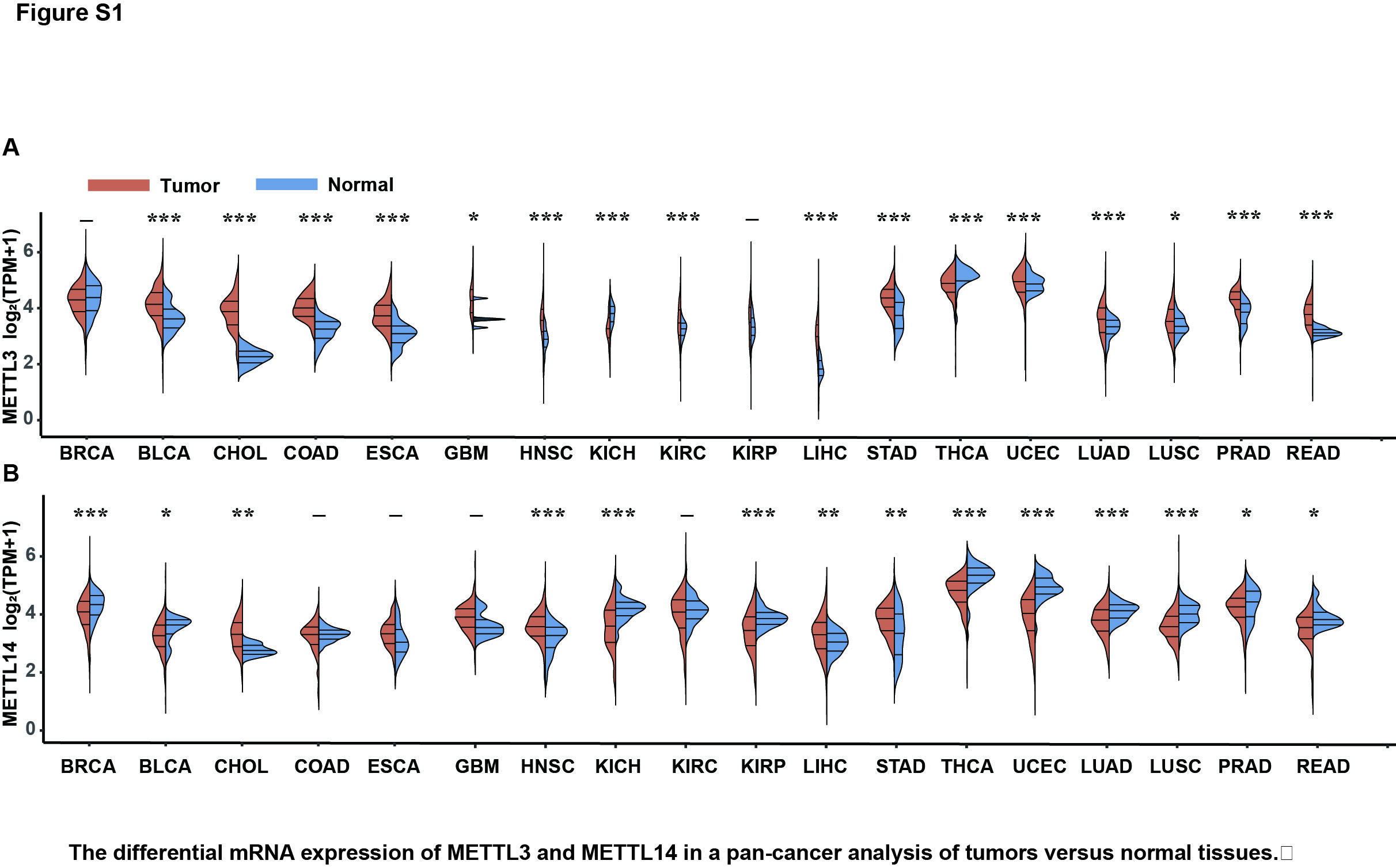

Supplement: Supplementary file 2 — Supplementary Figure S1. [file 41598_2024_64517_MOESM2_ESM.jpg]

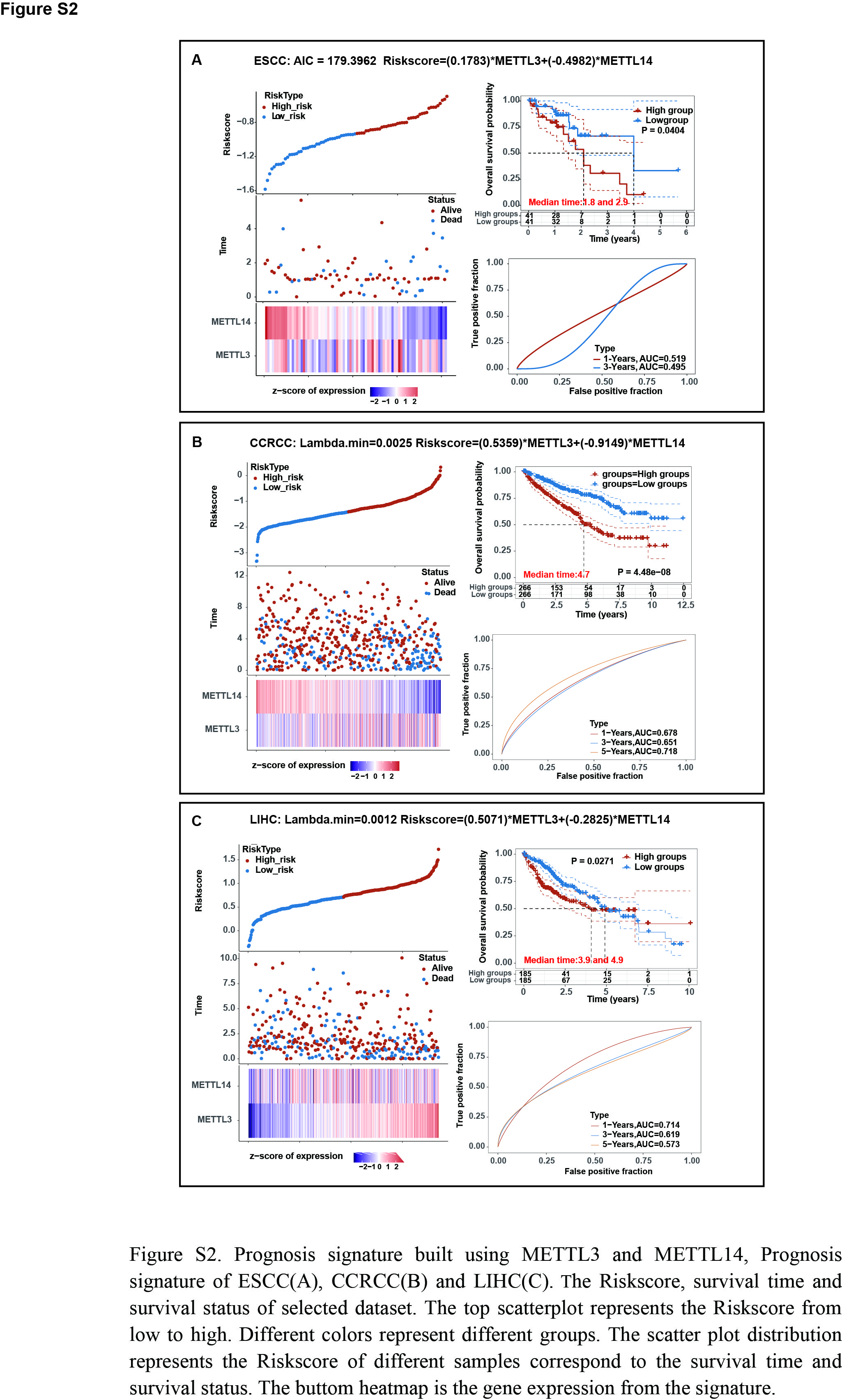

Supplement: Supplementary file 3 — Supplementary Figure S2. [file 41598_2024_64517_MOESM3_ESM.jpg]

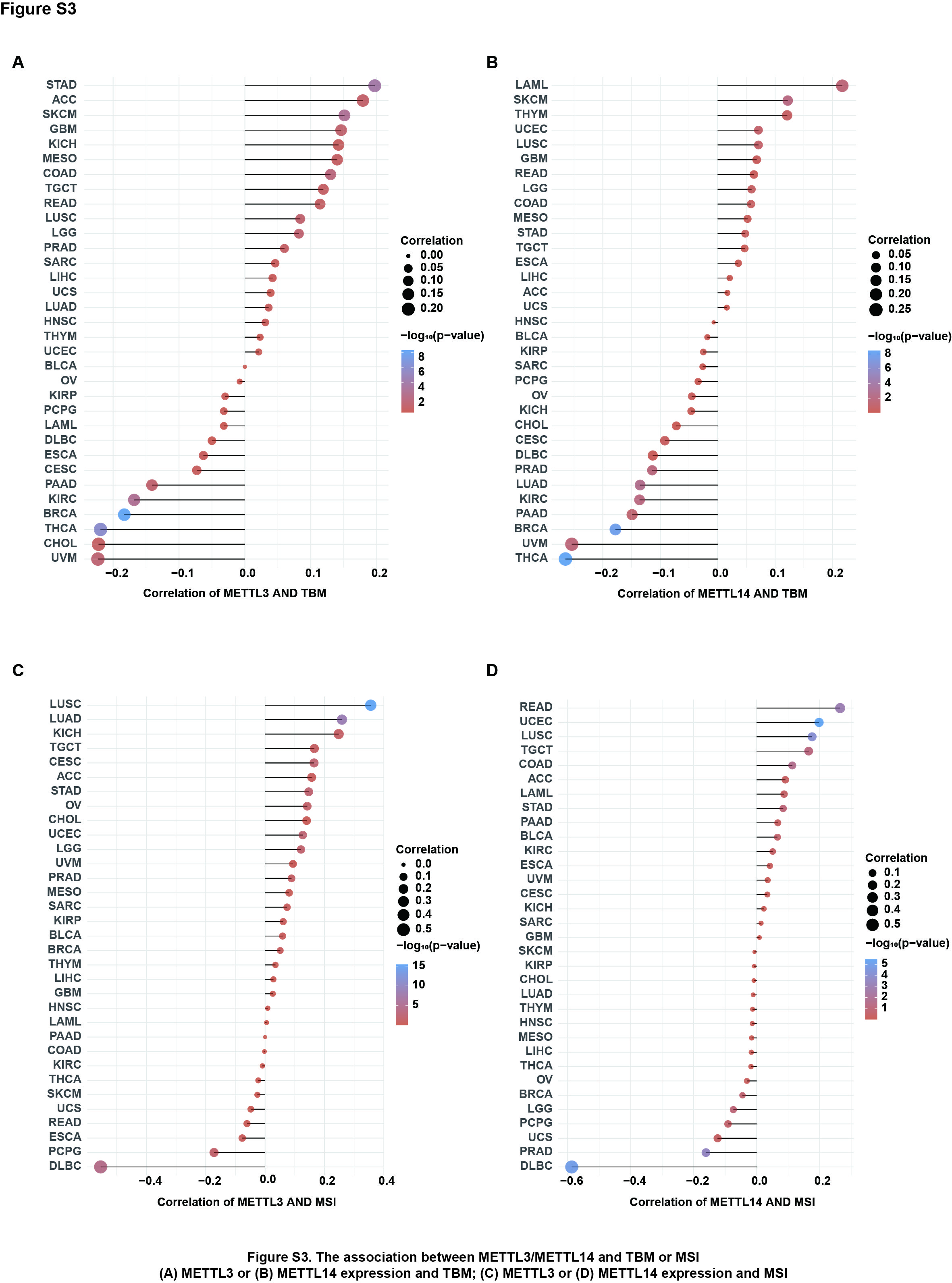

Supplement: Supplementary file 4 — Supplementary Figure S3. [file 41598_2024_64517_MOESM4_ESM.jpg]

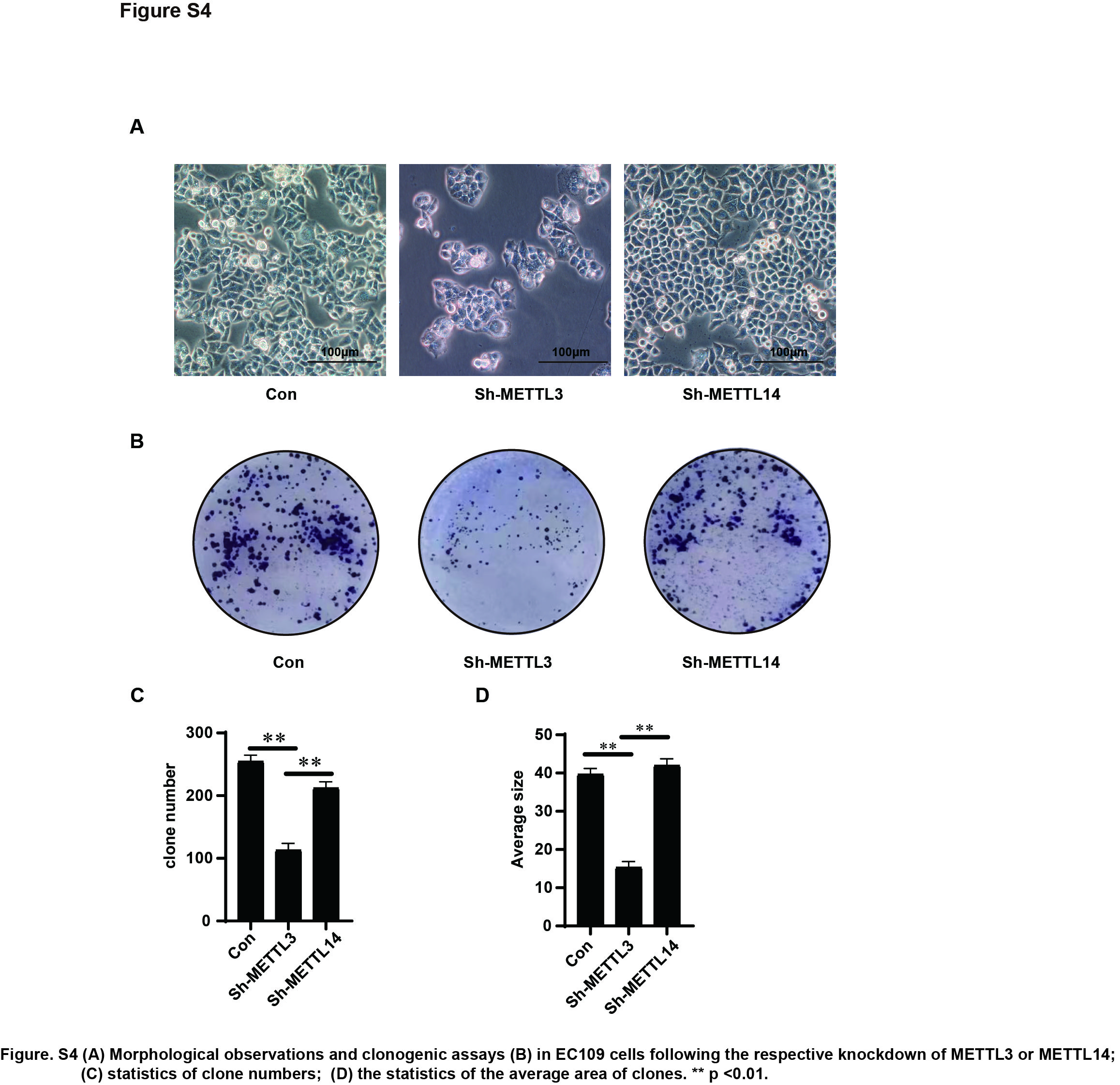

Supplement: Supplementary file 5 — Supplementary Figure S4. [file 41598_2024_64517_MOESM5_ESM.jpg]
